# Parathyroid hormone in Sri Lankan pregnant women: Vitamin D and other determinants

**DOI:** 10.1371/journal.pone.0258381

**Published:** 2021-10-08

**Authors:** Anusha Kaneshapillai, Usha Hettiaratchi, Shamini Prathapan, Guwani Liyanage

**Affiliations:** 1 Department of Biochemistry, Faculty of Medical Sciences, University of Sri Jayewardenepura, Nugegoda, Sri Lanka; 2 Department of Community Medicine, Faculty of Medical Sciences, University of Sri Jayewardenepura, Nugegoda, Sri Lanka; 3 Department of Paediatrics, Faculty of Medical Sciences, University of Sri Jayewardenepura, Nugegoda, Sri Lanka; Nanjing Medical University, CHINA

## Abstract

**Introduction:**

Determinants of parathyroid hormone level during pregnancy have been less frequently studied. We aimed to describe the serum parathyroid hormone (PTH) and its determinants in Sri Lankan pregnant women in a community setting.

**Materials and methods:**

In this cross-sectional analysis, 390 pregnant mothers in their third trimester were enrolled from primary care centers of 15 health divisions in the Colombo District in Sri Lanka. Venous blood was analyzed for a total 25-hydroxyvitamin-D [25(OH)D], serum parathyroid hormone (PTH), serum calcium, and alkaline phosphatase. The bone quality was assessed in terms of speed of sound (SOS) using the quantitative ultrasound scan (QUS). Univariate and multivariate regression analysis was used to examine the determinants of PTH concentration in blood.

**Results:**

Median serum 25(OH)D was 17.5ng/mL. Most (61.6%) were vitamin D deficient (<20ng/mL). Median PTH was 23.7pg/mL. Only 0.8% had hyperparathyroidism (PTH >65pg/mL). The correlation between 25(OH)D and PTH was weak but significant (r = -0.197; p<0.001). SOS Z-score was below the cut-off (≤−2) in fifty-six women (14.7%), and SOS did not relate significantly to PTH. In regression analysis, serum 25(OH)D, serum calcium, body mass index, educational level, and weeks of pregnancy were significant independent variables when adjusted. The model explained 16% of the variation in the PTH level.

**Conclusions:**

A high prevalence of vitamin D deficiency was observed among Sri Lankan pregnant women in the present study. Serum 25(OH)D, calcium, weeks of pregnancy, and educational level were determinants of serum PTH.

## Introduction

Calcium requirement increases during pregnancy. Parathyroid hormone (PTH) is vital for calcium homeostasis [1]. PTH has a very short half-life of 5 min and is influenced by subtle changes in serum calcium levels. Evidence support that PTH is involved in intestinal calcium absorption and maintaining serum ionized calcium levels through a feedback loop. In addition, in pregnant women, PTH regulates fetoplacental calcium homeostasis and skeletal development [2, 3]. Therefore, it is important to understand the determinants of PTH concentrations during pregnancy. The effect of dietary calcium, serum calcium, and 25(OH)D on PTH have been well studied [4, 5]. Yet, the available evidence on the effects of supplemental calcium on PTH is controversial in the background of lack of consensus regarding the recommended intake of calcium during pregnancy [6–8]. Previous reports suggest other determinants such as socioeconomic and lifestyle factors might predict PTH levels in non-pregnant adults, but research among pregnant women is lacking [9, 10].

Further, data bone quality using quantitative ultrasound (QUS) among pregnant women and its connection to PTH is sparse even though QUS is a useful, safe, and cheap tool [11, 12]. The comparison of dual-energy X-ray absorptiometry with QUS had shown a positive correlation between both methods. Therefore, QUS could be used to detect bone quality changes in pregnancy [13]. This study aimed to describe the vitamin D status and serum parathyroid hormone and its determinants in Sri Lankan pregnant women in a community setting.

## Materials and methods

### Study design and participants

We conducted this study in the Colombo District, located at a latitude of 6° 55’ 37’’ N, 9m above sea level, with a tropical climate (average temperature 27.5°C). There is an average of 2620 sunlight hours and an annual rainfall of 2500mm per year, without any distinct seasonal pattern [14]. A sample of 390 pregnant mothers in their third trimester (weeks of pregnancy ≥ 28weeks) from primary care centers in the Colombo District, from October 2018 to May 2019, was included in the study. These primary care centers are public-funded institutions. The registered pregnant women are followed up from the first trimester and referred to the local hospital for delivery [15]. The sample size was calculated, taking a ±5% margin of error, P of 63.4 [16], and a confidence level of 95%.

Recruitment was done with 2-stage stratified cluster sampling. There are 15 health divisions in the Colombo district. We randomly selected public-funded primary care clinics from health divisions, considering the population density of each. Different days were assigned (one day per week) for each clinic for subject recruitment. All those eligible women who attended the clinic on the designated day were invited to participate. We excluded women with multiple pregnancies, those using vitamin D or multivitamin supplements containing vitamin D, pregnant women taking medicines or suffering from diseases that may influence PTH metabolism, and those with severe pregnancy-induced complications or chronic medical conditions unrelated to pregnancy or bone disorders. Vitamin D supplementation is not a part of the nutrition protocol for pregnant women in Sri Lanka [15]. Therefore, women on ad hoc vitamin D supplementation were excluded. The study was carried out following the guidelines of the Declaration of Helsinki. The ethics review committee of the Faculty of Medical Sciences, University of Sri Jayewardenepura, approved the study protocol (ERC: USJP.FMS 20/18).

### Procedure

After obtaining written informed consent, obstetric history, past medical history, vitamin/mineral supplementation, diet, and socioeconomic background were logged. A panel of three experts in obstetrics and nutrition checked the content and face validity of the interviewer-administered questionnaire. Also, it was pre-tested among ten pregnant women from a community clinic for clarity of questions. Weight and height records at the first booking visit (8-12weeks) were taken from the clinic record to calculate the first-trimester body mass index. The total number of calcium tablets taken during the previous week was used to calculate oral calcium supplement intake per day. Also, dietary calcium and vitamin D intake were recorded using a food-frequency questionnaire. Food items rich in vitamin D and calcium were listed, portion sizes and frequency per week were noted. Estimation of dietary intake was performed with NutriSurvey 2007, modified for Sri Lankan food items and recipes [17]. The food items not indicated in the NutriSurvey 2007 were gathered from the Indian Food Composition Table, generated by the National Institute of Nutrition of Indian Council of Medical Research [18], and food labels in the local market.

They underwent a clinical examination following the initial interview, including anthropometric measurements (weight and height). In addition, two venous blood samples were collected for biochemical analysis between 8 a.m. and 12 noon: one sample for a total of 25(OH)D, calcium, inorganic phosphorous, and alkaline phosphatase (ALP) and another into an EDTA containing cold tube for PTH analysis. The serum was separated with cold centrifugation and stored at -80°C until biochemical analysis.

### Laboratory analysis and measurement of BMD

All biochemical analyses were done in the Department of Biochemistry at the University of Sri Jayewardenepura. LIAISON 25 OH Vitamin D TOTAL assay (DiaSorin Inc, Minnesota, USA) measured serum 25(OH)D and hydroxylated vitamin D metabolites with chemiluminescent immunoassay (CLIA). It has a limit of detection of 4.0ng/mL, inter and intra assay coefficient variation (CV) of 5.5–10.0%, and a negative bias of 15.3%. The LIAISON IN-TACT, PTH Gen II assay, was intended to quantitatively determine intact human PTH (DiaSorin Inc, Minnesota, USA). This assay measurement ranged from 3.0 and 1900pg/mL. The colorimetric method using Thermo Scientific Konelab 20XT analyzer measured calcium, inorganic phosphorous, and ALP.

Bone quality was measured with quantitative ultrasound (QUS) of the non-dominant tibia adhering to the manufacturer’s protocol (Sunlight Omni, Petah Tikva, Israel). QUS uses a gel as the coupling agent between the probe and the skin. The mechanism involves ultrasonic waves that travel along with the bones at a center frequency of 1.25 MHz. Axially transmitted speed of sound (SOS) (m/s) indicates overall bone quality by reflecting properties such as mineral density, elasticity, cortical thickness, and microstructure [19]. QUS is radiation-free and safe during pregnancy. Quality check was performed on each day before measuring. One trained investigator took all the QUS measurements using the same machine. The tibial measurement point (midpoint between the plantar surface of the foot and tip of the knee) was obtained in the seated position. Three scanning cycles were taken on each subject, keeping the leg horizontally using an ankle rest. Additional one/two cycles were taken if the information was not adequate as prompted by the machine. The Z-scores were calculated for speed of sound (SOS) according to the normative data provided by the manufacturer. The intra-operator co-efficient variation (CV) for this population was 4.3%.

### Definitions

Vitamin D levels were defined as deficient (<20ng/mL) and insufficient (20-29ng/mL) [20]. The severe deficiency was defined as <10ng/mL. The reference ranges considered were total serum calcium (normal 2.15–2.51 mmol/L) [21], PTH (10–65 pg/mL), bone-specific ALP (30–125 U/L) [22], inorganic phosphorus (0.87–1.45 mmol/L) [23]. Recommended level of dietary calcium and vitamin D intake for both pregnant and lactating populations was 1000 mg/day and 600 IU/day [24]. Low bone quality was defined at SOS, Z-scores ≤−2 using the manufacturer’s reference values. Employment status was classified as employed in income-generating activity or housework. Educational status was classified as less than secondary (up to grade 11 or less), secondary education (up to grade 13), and post-secondary education (diploma/degree or higher).

### Statistical analysis

SPSS software package was used for data analysis. For all the tests performed, the level of significance was taken as 0.05. Continuous variables were expressed as mean with standard deviation or the median with an interquartile range. The categorical variables were expressed as frequencies and percentages. Man-Whitney U test was performed to examine the serum PTH and SOS-Z scores between vitamin D deficient and sufficient groups. Spearman correlation was performed to examine the correlation between skewed data, where applicable. To examine the association of PTH with potential predictors, univariate and multivariate linear regression analysis was carried out. After confirming normality, the logarithm (Log10) of PTH and 25(OH)D was used for this purpose. With prior knowledge, the following independent variables were considered for simple linear regression: age, parity, educational level of the respondents (less than secondary or secondary or post-secondary), ethnicity, employment status, household income (≥57,000 vs. <57,000 LKR), weeks of pregnancy at recruitment, first trimester BMI, third trimester BMI, dietary and supplemental calcium intake, dietary vitamin D intake, bone quality, serum calcium, and bone-specific alkaline phosphatase level [5.6]. Independent variables were examined for multicollinearity with correlation coefficients and variance inflation factor (VIF). Coefficients of >0.80 or VIF value >3 were excluded. Subsequently, multiple linear regression was performed using all potential predictors with significant p values (<0.05) and important variables through literature search (educational level as a proxy variable for socioeconomic status).

## Results

### Baseline characteristics of the study sample

A total of 650 pregnancy records were screened, and 428 were eligible. Reasons for non-eligibility were not in the third trimester, receiving vitamin D supplements, and chronic diseases and pregnancy-related complications. Subsequently, 428 eligible pregnant women were invited. Of them, 390 subjects were enrolled. The response rate was 91.1%. Refusal of consent, incomplete questionnaires, and haemolysed blood samples were reasons for dropouts. Table 1 gives the baseline characteristics. The mean age at blood draw was 28.9±5.7 years. Most women had either secondary or post-secondary (90.3%). The majority (59.5%) were on calcium (dietary and supplemental) of >1000 mg/day. However, most took <600IU of vitamin D in their diet (61%), none of them were on vitamin D supplements. Median serum 25(OH)D was 17.5ng/mL. The prevalence of vitamin D deficiency was 61.6% (<20ng/mL as specified in the diagnostic criteria). Forty-one women (10.5%) had severe deficiency (<10ng/mL) and 33.1% had insufficiency. Median PTH was low (23.7pg/ml). Only three pregnant women had hyperparathyroidism (defined as >65pg/mL). Seven (1.8%) had serum calcium less than the lower limit of normal. Bone-specific ALP was high among 4.8% (n = 19). Fifty-six (14.7%) had low SOS Z scores indicating low bone quality (SOS, Z-scores ≤−2).

**Table 1:** Baseline characteristics of the study sample (n = 393).

Characteristic	Measurement
Age (years), mean (SD)	28.90 (5.72)
Total family income (LKR), median (IQR)	40,000 (55,000, 30,000)
Education	
Less than secondary	36 (9.3)
Secondary	302 (77.4)
Post-secondary	52 (13.3)
Employment status	
Housework	301 (76.6)
Income-generating	92 (23.4)
Ethnicity	
Sinhalese	296 (75.3)
Tamil	43 (10.9)
Moor	54 (13.7)
Weeks of pregnancy, mean (SD)	32.95 (3.54)
First trimester BMI (kg), mean (SD)	23.64 (4.79)
Third trimester BMI (kg), mean (SD)	26.8 (4.64)
Total calcium intake (mg/day), mean (SD)	1122 (400)
Dietary vitamin D intake (IU/day), median (IQR)	450.0 (1218, 272)
Parity, median (IQR)	1 (0, 1)
PTH (pg/mL) (n = 388), median (IQR)	23.7 (18.4, 30.7)
25(OH)D (ng/mL), median (IQR)	17.9 (13.1, 22.6)
Serum calcium (mmol/L), median (IQR)	2.38 (2.32, 2.42)
Bone specific ALP (U/L), median (IQR)	74 (94, 61)
*SOS: Z scores (n = 381), median (IQR)	-0.5 (-1.5, 0.6)

Abbreviations: PTH: Parathyroid hormone; ALP: Alkaline phosphatase; BMI: Body mass index, LKR: Sri Lankan rupees; SD: Standard deviation, SOS: speed of sound.

### Relationship of PTH with vitamin D and other potential predictors

A significant inverse relationship was observed between 25(OH)D and PTH; however, the correlation coefficient was low (r = -0.197; p<0.001). Deficient women (<20ng/ml) had a higher PTH level than the sufficient women; the difference was statistically significant (U = 1.397, p = 0.001). Serum 25(OH) D did not correlate significantly with serum calcium (p = 0.076) or alkaline phosphatase (p = 0.973). Serum vitamin D did not have a significant relationship to dietary vitamin D intake (p = 0.793) or calcium intake (supplemental and dietary) (p = 0.667). PTH showed a significant correlation with serum calcium (r = -0.345, p = 0.001), and bone-specific alkaline phosphatase (r = -0.139, p = 0.006). However, PTH did not correlate considerably with total calcium or vitamin D intake (p>0.05). The SOS Z-scores did not have a significant correlation with serum calcium, ALP, 25 (OH)D, PTH, total calcium intake, and vitamin D intake in the diet (p>0.05). Also, SOS-Z scores were not significantly different among vitamin D deficient (<20ng/ml) and sufficient groups (U = 16536, p = 0.867).

A significant but weak correlation was observed between weeks and pregnancy and ALP (r = 0.220, p<0.001) and between weeks of pregnancy and PTH (r = 0.114, p = 0.025). Women with secondary and post-secondary had lower BMI compared to women with less than secondary education (OR = -0.151; CI = -3.21, -0.11; p = 0.036 and OR = -0.159; CI = -4.10, -0.249; p = 0.027). However, total family income was not related to BMI (p = 0.763).

### Determinants of log10 PTH

In univariate regression, log10 PTH was positively associated with weeks of pregnancy and ethnicity (Moor compared to Sinhalese), whereas it was inversely associated with serum 25(OH)D and total serum calcium (Table 2). Statistically significant (p<0.05) variables and important variables through literature search (viz. educational level as a proxy variable for socioeconomic status) were considered for inclusion in the model. Bone-specific ALP and first trimester BMI were removed as 95% confidence interval (CI) included zero. Ethnicity did not contribute to the model significantly. Table 3 describes the outcome of the multiple linear regression. The model showed that PTH was lowered with increasing serum calcium and serum 25(OH)D. The effect size was highest with serum calcium, followed by 25(OH)D. Women with post-secondary education had lower odds of having high PTH than women with primary education. The model explained 21% of the variation in the PTH level.

**Table 2:** Determinants of Log PTH (transformed PTH) concentrations in univariate analysis.

Characteristic	Standardized Coefficients (B)	95% Confidence Interval for B		p-value
Age (years)	-0.002	-0.005	-0.001	0.199
Education				
Less than secondary	Ref			
Secondary	-0.026	-0.071	-0.018	0.241
Post-secondary	-0.021	-0.076	-0.034	0.446
Employment status				
Housework	Ref			
Income-generating	-0.018	-0.062	0.026	0.420
Total family income >57,000 LKR	0.006	-0.039	0.050	0.797
Ethnicity				
Sinhalese	Ref			
Tamil	0.058	0.002	0.117	0.052
Moor	0.076	0.021	0.130	0.007
Weeks of pregnancy	0.006	0.001	0.011	0.027
First trimester BMI (kg)	0.004	-0.000	0.008	0.044
Third trimester BMI (kg)	0.005	0.001	0.009	0.027
Total calcium intake (mg/day)	-0.002	-0.007	0.003	0.380
Dietary vitamin D intake (IU/day)	-0.001	-0.003	0.001	0.266
Parity	0.018	-0.006	0.042	0.135
Log _10_ 25(OH)D (ng/mL)	-0.238	-0345	-0.131	<0.001
Serum total calcium (mmol/L)	-0.579	-0.742	-0.416	<0.001
Bone specific ALP (U/L)	0.001	0.000	0.002	0.006
*SOS: Z scores	-0.006	-0.017	0.006	0.308

Abbreviations: ALP: Alkaline phosphatase; BMI: Body mass index, LKR: Sri Lankan rupees; SD: Standard deviation, SOS: speed of sound.

**Table 3:** Determinants of Log_10_ PTH (transformed PTH) concentrations in the final model.

	Standardized Coefficients (B)	95% Confidence Interval for B		P-value
		Lower	Upper	
Log_10_ 25(OH)D (ng/mL)	-0.215	-0.314	-0.116	<0.001
Serum total calcium (mmol/L)	-0.624	-0.783	-0.465	<0.001
Weeks of pregnancy	0.007	0.002	0.012	0.004
Education				
Less than secondary	Ref			
Secondary	-0.056	-0.113	-0.001	0.052
Post-secondary	-0.086	-0.157	-0.015	0.018
BMI (Third trimester)	0.006	0.002	0.010	0.001

Overall model (F 6,392 = 16.76; p<0.001; R^2^ = 0.21).

## Discussion

The prevalence of vitamin D deficiency was 62% among the pregnant women in the Colombo District in Sri Lanka, where vitamin D supplementation is not a component of the national nutrition protocol and sunshine is abundant throughout the year. In brief, women in this cohort were average gestation of 33 weeks, recruited from public-funded primary care clinics of 15 health divisions. They were healthy without pregnancy-induced complications, chronic medical conditions, or bone disorders; they were on calcium lactate and iron sulfate supplements.

We confirmed a significant but weak inverse relationship between 25(OH)D and intact PTH, which agrees with the findings of previous studies [25–27]. The deficient women had higher mean PTH concentrations than the non-deficient. Yet, they did not demonstrate hyperparathyroidism, defined as PTH >65pg/mL. Therefore, it could be speculated that there are additional determinants of serum PTH concentrations other than serum 25(OH)D [5, 6].

Determinants of PTH levels during pregnancy had not been investigated in Sri Lankan pregnant women previously. In the current study, serum 25(OH)D, serum calcium, educational level, and weeks of pregnancy were statistically significant predictors of PTH in the multivariate model. Serum calcium was inversely associated with PTH and showed the highest association. It supports the previous evidence of PTH involvement in feto-maternal calcium homeostasis [2, 3]. Interestingly, serum PTH had a significant but weakly positive correlation to weeks of pregnancy, and it is in line with previous longitudinal studies; serum PTH level falls to the low-normal range during the first trimester (10–30% of the mean non-pregnant value), and after that, it increases steadily to a mid-normal range by the term [2]. The demand for calcium for the fetus increases towards the third trimester. Therefore, it can be speculated that increasing PTH towards the third trimester may be responsible for higher calcium mobilization to support the needs of the growing fetus [28].

The total calcium intake (diet and supplements) and vitamin D intake (diet) were less than recommended in 40% and 61% of women in the present study [24]. In our analysis, a significant impact of calcium and vitamin D intake on PTH concentration was not demonstrated. However, varying effects of dietary and supplemental calcium intake on PTH concentration are described previously [8, 9]. The dietary or supplemental intake did not affect PTH among healthy, Caucasian pregnant women from Ireland [8]. In contrast, a study among postmenopausal women reported that calcium intake (1000mg/day) and vitamin D intake (1000IU/day) suppressed PTH significantly; still, neither calcium nor vitamin D supplements alone suppressed serum PTH [9]. The mixed results could be attributed to a lack of homogeneity and design errors. Recall bias affects the assessment of dietary and supplemental calcium intake through food frequency questionnaires. Also, it could be argued that a recall questionnaire does not necessarily reflect the usual calcium intake. Further, lack of food-list updates, particularly local food items and inaccuracies of portion sizes, are other possible reasons.

The relationship between body fat content and PTH had been well studied in the general population but limited among pregnant women [5, 29–31]. Serum PTH plays an important role in fat accumulation through several mechanisms, including its effect through vitamin D [30]. Yet, Green et al. failed to demonstrate a connection between BMI and PTH [5]. In the present study, BMI demonstrated a significant but weak association with PTH levels.

The educational level was linked to serum PTH concentration. Women with post-secondary education had lower PTH concentration than women with less than secondary education. This association could have been indirectly influenced through BMI as the women with secondary and post-secondary education had higher odds of increased body mass index than women with post-secondary education [32, 33]. This inverse relationship between PTH and social inequalities (viz. educational level) has been reported previously by Navarro Mdel et al. among post-menopausal women [32].

Due to the high calcium demand in pregnancy, bone turnover is high [34]. Therefore, regardless of the counter-regulatory mechanisms, bone mineral density declines about 3% during pregnancy [34]. Previous reports have suggested that bone mineral density is influenced by PTH, growth hormone, prolactin, estrogen, nutritional habits, body weight, and lifestyle [35]. Therefore, we intended to evaluate whether bone quality is a determinant of PTH. Yet, we could not demonstrate a relationship between the two variables. A likely explanation is the low accuracy of QUS compared to other methods. Recent evidence supports that QUS is a reasonable indicator of bone loss in non-pregnant young women [36]. It is safe, less expensive, and easy to use. However, dual-energy X-ray absorptiometry (DEXA) is considered the “gold standard” test for bone mineral density [37]. Although earlier models of DEXA scans were not considered safe during pregnancy, the introduction of fan-beam densitometers that emit low ionizing radiation are safe to use from the beginning of the second trimester of pregnancy. Yet, they are expensive and not widely available in resource-poor settings.

The results of this study should be interpreted with the following limitations. First, the cross-sectional study design is a limitation, and it generally does not confirm causation. Second, most of the women were housewives and from low- and middle-income families. Thus, results cannot be generalized to all socioeconomic strata. Finally, both DEXA scan and QUS together instead of QUS alone could have evaluated bone mineral density and quality more reliably.

## Conclusions

A high prevalence of vitamin D deficiency was observed in the present study among Sri Lankan pregnant women. Serum 25(OH)D, calcium, weeks of pregnancy, and educational levels were determinants of serum PTH.

## Supporting information

S1 Questionnaire Data collection sheet. (PDF)
