# Supplementary material for: Parathyroid hormone in Sri Lankan pregnant women: Vitamin D and other determinants
Source: PLoS One. 2021 Oct 8;16(10):e0258381. doi: 10.1371/journal.pone.0258381 (PMC8500411; doi:10.1371/journal.pone.0258381)
Supplement: S1 Questionnaire — (PDF) [file pone.0258381.s001.pdf]

Date: 08/05/2018 Version 1.0

No……………

Data collection sheet

Section I

**01**	**MOH area**	
**02**	**Sector**	Urban Rural Estate
**04**	**Date of birth**	Y M D .... / Age ....
**05**	**Permanat address**	
**06**	**Contact No**	Mobile :<br>Land :
**07**	**Ethnicity**	Tamil Sinhala Moors Burgher
**08**	**Employed (income generating)**	Yes/No
**09**	**Level of educational**	≤ Grade 11<br>Up to grade 13<br>post-secondary non-tertiary<br>Tertiary
**10**	**Monthly income of the family**	
**11**	**Medical and surgical history (past)**	Diabetes ☐<br>Hypertension ☐<br>Congenital/acquired heart disease ☐<br>Anaemia ☐<br>Other haematological disorders: ☐<br>Bone disorders ☐
**12**	**Pregnancy related medical issues**	Diabetes ☐<br>Hypertension ☐<br>Others:
**13**	**Gestation at recruitment**	Ultrasound based:<br>Calculated by LRMP:
**14**	**Parity**	

			Duration	Dosage
**15**	**Currently taking nutrient supplements**	Iron		
		Folic acid		
		Calcium		
		Any other		

**16**	**Height (cm)**	
	**Weight (Kg) current**	
	**Weight at the booking visit**	
**17**	**Gestation at the booking visit**	

Date: 08/05/2018 Version 1.0

No…………….

Section II

Food frequency

Food	Portion size	No of portions at each meal	Total portions/day	Total portions/week
**Milk powder** (specify the brand name)				
**Fresh milk** specify the brand name				
**Other milk products**				
Cheese				
Yoghurt				
Butter				
Margarine				
Curd				
**Small fish**				
Sardinella (Salaya)				
Sardinella (Hurulla)				
Kaaralla				
Anchovy fish				
Sprats				
Others				
**Large fish**				
Tuna (balaya)				
Yellow fin tuna (kelavalla)				
Sail fish (thalapath)				
Mackerel (Kumbalawaa)				
Salmon (saman)				
Others				
**Eggs**				
**Meat and meat products**				
**Fruits**				
Papaya				
guava				
watermelon				
mango				
banana				
avocado				
oranges				
Nelli				
Others				

**Green leaves**				
Drumstick leaves				
Cauliflower leaves				
Pumpkin leaves				
Other green leaves				
**Vegetables**				
Broccoli				
Cauliflower				
Mushrooms				
Carrots				
Okra/ladies finger				
Soybean				
Others				
**Other food items other than mentioned above**				
**Other beverage items other than mentioned above**				
